# Serum Immune-Related Proteins are Differentially Expressed during Hibernation in the American Black Bear

**DOI:** 10.1371/journal.pone.0066119

**Published:** 2013-06-25

**Authors:** Brian A. Chow, Seth W. Donahue, Michael R. Vaughan, Brendan McConkey, Mathilakath M. Vijayan

**Affiliations:** 1 Department of Biology, University of Waterloo, Waterloo, Ontario, Canada; 2 Department of Mechanical Engineering, Colorado State University, Fort Collins, Colorado, United States of America; 3 Department of Fish and Wildlife Conservation, Virginia Polytechnic Institute and State University, Blacksburg, Virginia, United States of America; New Jersey Medical School, University of Medicine and Dentistry of New Jersey, United States of America

## Abstract

Hibernation is an adaptation to conserve energy in the face of extreme environmental conditions and low food availability that has risen in several animal phyla. This phenomenon is characterized by reduced metabolic rate (∼25% of the active basal metabolic rate in hibernating bears) and energy demand, while other physiological adjustments are far from clear. The profiling of the serum proteome of the American black bear (*Ursus americanus*) may reveal specific proteins that are differentially modulated by hibernation, and provide insight into the remarkable physiological adaptations that characterize ursid hibernation. In this study, we used differential gel electrophoresis (DIGE) analysis, liquid chromatography coupled to tandem mass spectrometry, and subsequent MASCOT analysis of the mass spectra to identify candidate proteins that are differentially expressed during hibernation in captive black bears. Seventy serum proteins were identified as changing by ±1.5 fold or more, out of which 34 proteins increased expression during hibernation. The majority of identified proteins are involved in immune system processes. These included α_2_-macroglobulin, complement components C1s and C4, immunoglobulin μ and J chains, clusterin, haptoglobin, C4b binding protein, kininogen 1, α_2_-HS-glycoprotein, and apoplipoproteins A-I and A-IV. Differential expression of a subset of these proteins identified by proteomic analysis was also confirmed by immunodetection. We propose that the observed serum protein changes contribute to the maintenance of the hibernation phenotype and health, including increased capacities for bone maintenance and wound healing during hibernation in bears.

## Introduction

Hibernation is an adaptation to cope with extreme environmental conditions and low food availability [1]. This process is characterized by changes in the homeostatic set points of the organism, including body temperature, leading to the depression of the metabolic rate and a corresponding decrease in energy demand. While the molecular bases of hibernation in small mammals and ectotherms are beginning to be understood (see review [1]), fewer studies have been carried out on such molecular adaptations to hibernation in ursids [2].

Ursids, including the American black bear (*Ursus americanus*), are among the largest animals that hibernate, and exhibit some of the typical physiological and biochemical changes common amongst hibernating animals, including lowered body temperature and metabolic rate [3], [4], slowed heart rate [3], altered serum composition [5], and the catabolism of lipids as a primary energy source [6]. In contrast to small, “deep” hibernating mammals, including sciurids, the core body temperature of hibernating ursids is only decreased by a few degrees Celsius and there is a lack of frequent arousals [1]. Also, the metabolic rate, as a percentage of the active basal metabolic rate, is depressed to ∼25% in hibernating black bear compared to ∼2–5% in deep hibernators [4]. There appears to be striking and unique changes in ursid metabolism during hibernation, including the near complete conservation of nitrogen [6] and the maintenance of wound healing [7]. As well, hibernating bears prevent disuse osteoporosis by maintaining balanced bone resorption and formation [8], [9], [10]. While the factors contributing to the hibernation phenotype is not clear, changes in unidentified serum components have been reported with hibernation in bears previously. In contrast, some small hibernators exhibit decreased protein translation [11], suppression of wound healing [12], [13], and an imbalance in bone remodeling, leading to loss of bone mass [14], [15]. Consequently, research into the regulatory mechanisms allowing for such unique hibernation phenotype in bears may yield insights into treatments for human diseases [16].

A recent study demonstrated that broad changes in gene expression patterns, rather than specific hibernation-related genes, reflect changes in metabolism during hibernation in black bears [17], similar to that seen in small mammals [11], [18]. The gene expression changes may give rise to the remodeling of tissue proteome that may be essential for hibernation in bears. Indeed protein turnover is elevated in black bear serum during hibernation [17], [19], and changes in specific serum proteins, including acute phase proteins [20], [21] and aminotransferases [22] have been reported in hibernating bears. However, systemic changes in the serum proteomes of large hibernators, including bears, have not been characterized to our knowledge. The objective of this study was to examine the changes in the serum proteome of the active and hibernating black bear to identify differentially expressed proteins in order to provide novel insights into the biochemical adaptation to hibernation in bears. The serum proteome changes were assessed using a two-dimensional difference gel electrophoresis (DIGE) approach [23] from the same animal prior to and during hibernation. A subset of the proteins identified by DIGE were also confirmed to be differentially regulated using SDS PAGE followed by immunodetection.

## Materials and Methods

### Animals

For the comparison of serum proteomes between active and hibernating black bears, paired serum samples from 8 animals (animals 1–8 in Table 1) were taken from two time points: prior to (PRE) and during hibernation (HIB). For the follow up western immunoblotting studies, paired serum samples were used from an additional four animals (Table 1). Animals were anesthetized with a 2∶1 mixture of ketamine (100 mg/ml)∶xylazine (100 mg/ml) at a dosage of 1 cc of the mixture per 45.5 kg of body mass. Behavior indicating stress was not observed during any of the handling procedures. Blood samples were drawn from the femoral vein while the animal was anesthetized, and the samples were transported to the laboratory in an ice- packed cooler. Immediately on return to the laboratory, the blood was spun to isolate the serum and was frozen at −20°C. The Virginia Polytechnic Institute and State University Animal Care Committee approved all bear handling protocols (#98-069- F&WS).

**Table 1:** Black bear sample information.

Bear #	Sampling Days for PRE and HIB samples, respectively (Julian Day)	Pregnant at Time of Den Entry	Parturition Day (Julian Day)	Initial Weight (lbs)	Weight Change during Hibernation (% of Initial)
1	324, 59	Y	14	186	−20.9%
2	334, 49	N	NA	186	−20.5%
3	283, 69	Y	28	181	−24.9%
4	324, 69	Y	11	256	−20.2%
5	294, 49	Y	2	111	−34.3%
6	285, 49	N	NA	93	−40.4%
7	276, 59	Y	36	156	−27.1%
8	284, 59	N	NA	186	−23.8%

### Difference gel electrophoresis (DIGE)

Black bear sera were processed prior to proteome analysis exactly as described previously for grizzly bear sera [24]. Briefly, serum samples were depleted of albumin and IgG with Aurum Serum Protein Mini-kits (BioRad, Hercules, CA, USA), and total serum protein concentration was determined by the bicinchoninic method [25] using bovine serum albumin (Thermofisher Scientific, Waltham, MA, USA) as the standard. After processing, proteins were separated by a modified DIGE method [23]. Proteins were precipitated using a 2-D Clean-Up kit (GE Healthcare, Piscataway, NJ, USA) and resuspended to a final concentration of 5 µg protein/µL in lysis buffer [7 M urea, 2 M thiourea, 5 mM magnesium acetate, 30 mM TRIS, 4% (v/v) CHAPS; all reagents purchased from Thermofisher Scientific]. Samples were labeled with CyDye DIGE Fluor minimal dyes (GE Healthcare) according to manufacturer's instructions and to minimize dye bias. A pooled internal standard consisting of 25 µL of each sample was labeled with Cy2 dye. 50 µL of labeled pre-hibernation serum samples were combined with 50 µL of hibernation samples and 50 µL of the internal standard. Each of these combined samples were diluted in rehydration buffer [7 M urea, 2 M thiourea, 4% (v/v) CHAPS, 40 mM DTT, 0.5% (v/v) pH 4–7 IPG buffer] and rehydration-loaded onto 24 cm, pH 4–7 Immobiline DryStrip IPG strips (GE Healthcare) in a reswelling tray for 12 h. Isoelectric focusing was done on a IPGphor II (GE Healthcare) under the following conditions: step 100 V, 1 h; step 500 V, 2 h; gradient to 1000 V, 2 h; gradient to 3000 V, 3 h; step 3000 V, 2 h; gradient to 8000 V, 3 h; step 8000 V, 9 h; step to 500 V, 13 h; total 109600 Vh. For the second dimension separation, IPG strips were equilibrated [6 M urea, 30% (v/v) glycerol, 50 mM TRIS, bromophenol blue (Thermofisher Scientific)] with first 1% (w/v) DTT for 30 min, then 2.5% (w/v) IAA for an additional 30 min. Equilibrated strips were placed on top of 12% SDS-PAGE gels and sealed with 1% agarose. 2^nd^ dimension electrophoresis was done using an Ettan DALTsix electrophoresis unit (GE Healthcare) at 1.5 W per gel for 30 min, then 17.5 W per gel until the dye front reached the bottom of the gels. Gels were scanned on a Typhoon Variable Mode Imager (GE Healthcare) at excitation/emission wavelengths of 457/520 nm (Cy2), 532/580 nm (Cy3), and 633/670 nm (Cy5). Protein spot expression was analyzed with DyCyder 7 software (GE Healthcare). After spot expression analysis, a preparatory gel was run to isolate proteins for identification by mass spectrometry. 1st dimension separation was the same as described above, except proteins were not labeled with CyDyes, and 500 µg unlabeled, pooled serum protein was loaded onto a single IPG gel strip. 2nd dimension separation was the same as described above. After 2D separation, the gel was stained with colloidal Coomassie G-250 [0.12% (w/v) Coomassie G-250, 10% (w/v) ammonium sulfate, 10% (v/v) phosphoric acid, 20% (v/v) methanol] and destained with 10% (v/v) phosphoric acid and 20% (v/v) methanol, then dH_2_O. Protein spots of interest were excised manually and stored in microcentrifuge tubes at 4°C with deionized water until mass spectrometric analysis.

### Tandem mass spectrometry (MS/MS)

Protein spots were identified by tandem mass spectrometry using a Qtrap 2000 LC/MS/MS (Applied Biosystems, Foster City, CA). A gel piece containing 1 pmol bovine serum albumin (BSA) stained with colloidal Coomassie stain was digested in parallel with protein spots as a control. Excised gel plugs were diced into approximately 1 mm^3^ portions, and the Coomassie stain was removed by washing three times with ddH_2_O, three times with 50 mM NH_4_CO_3_ in 50% acetonitrile (ACN), and a final wash with 100% ACN for 5 min each (Thermofisher Scientific). After washing, samples were reduced with 10 mM DTT in 100 mM NH_4_CO_3_ for 30 min at 50°C. After another 5 min 100% ACN wash, samples were alkylated with 55 mM IAA in 100 mM NH_4_CO_3_ for 30 min at room temperature in the dark. Samples were washed again with 100 mM NH_4_CO_3_ for 15 min, then 100% ACN for 5 min. Samples were dried down on a SpeedVac (Thermofisher) at 4°C for 20 min. 10 ng trypsin in 100 mM NH_4_CO_3_ was added to the samples and incubated for 16 h in a 37°C water bath. Samples were diluted with ddH_2_O and bath sonicated for 10 min. After centrifugation at 1000 rpm for 30 s, the supernatant was transferred to a collection tube with 5 uL of 5% formic acid (FA) in 50% ACN. Digested peptides remaining in the digested gel plugs were extracted with 5% formic acid (FA) in 50% ACN. The volume of the collected supernatant was reduced to 10 to 15 uL in a SpeedVac. Samples were cleaned using C18 ZipTips (Millipore, Billerica, MA, USA). Samples were acidified with 1% FA, and peptides were bound to equilibrated ZipTips (wetting with 50% ACN 3 times, then 0.1% FA 3 times) by 20 cycles of drawing and expelling of the sample. ZipTips were washed twice with 0.1% FA, and peptides were eluted by 10 cycles of drawing and expelling of 5 uL 50% ACN. Peptides were identified using MASCOT MS/MS Ion Search (Matrix Science, Boston, Massachusetts, USA) against the NCBI non-redundant protein database. Mass spectra with fewer than 30 peaks were discarded, except for very dilute samples where we discarded spectra with fewer than 10 peaks. The following parameters were used for MASCOT searches: trypsin digestion, carbamindomethyl fixed modifications, methionine oxidation variable modifications, monoisotropic mass values, ±1.2 Da peptide and ±0.8 Da fragment mass tolerances, and up to one missed cleavage. GOMiner was used to determine significantly enriched Gene Ontology (GO) pathways [26]. UniProt gene identifiers for homologous human genes were obtained for each unique, differentially expressed black bear protein (n = 15) that was identified by MS/MS. A list of the N-terminal serum proteome of human blood (n = 213) was used as the background total gene list [27] because similar data does not exist for the American black bear. The enrichment of each GO category was calculated as the proportion of changed to total proteins for each category. GO categories with high enrichment (>1.5) was ascertained by one-tailed Fisher's exact test and p-values<0.05 were considered significant.

### Western blotting

Commercial antibodies against several proteins were obtained, including sheep anti-human α-2-macroglobulin (Affinity BioReagents, Golden, Colorado, USA), goat anti-dog transferrin (Tf, Bethyl Laboratories, Montgomery, Texas, USA), sheep anti-human apolipoprotein A1 (ApoA1, Abcam, Cambridge, Massachusetts, USA), rabbit anti-human haptoglobin (Hp, Sigma, St. Louis, Missouri, USA), anti-rabbit α1-antitrypsin (A1AT, Sigma), and anti-rabbit kininogen 1 (KNG, Sigma). Secondary antibodies were also obtained, including donkey anti-sheep IgG and rabbit anti-goat IgG (Bethyl), and goat anti-rabbit IgG (BioRad), all conjugated to horseradish peroxidase (HRP). Serum proteins were detected by western immunoblotting exactly as described previously [24]. Total protein concentrations were determined by the BCA method [25], and samples were adjusted to 125 ug protein/mL with Laemmli's buffer [28]. 2.5 µg of total protein was loaded into wells on polyacrylamide gels along with a broad range molecular weight protein standard (BioRad), and proteins were separated at 200 V for 45 min using a discontinuous buffer. Proteins were transferred to a 0.22 µm pore size membrane (BioRad) using a TransBlot SD semi-dry electrophoretic transfer cell (BioRad). Transfer efficiency was checked by Ponceau S staining of the membrane. Membranes were then washed with Tris buffered saline with Tween-20 [TTBS; 20 mM Tris, 300 mM NaCl, 0.1% Tween-20 (BioShop, Burlington, ON, Canada), pH 7.4] and blocked with 5% skim milk powder in TTBS (SM) for 1 h at room temperature. Blots were rinsed with TTBS, and 10 mL primary detection antibody diluted 1∶1500 in SM was added. After 1 h incubation, blots were washed 3× with TTBS, and appropriate secondary antibody diluted 1∶3000 in SM was added. After another 1 h incubation, membranes were washed 3× with TTBS and 1× with TTBS without Tween-20. ECL Plus (GE Healthcare) detection solution was freshly prepared, and 1 mL was applied to the membrane. After 5 min, membranes were scanned on a Pharos scanner using the QuanityOne software (BioRad). Protein band densitometry was performed using ImageJ 1.45s [29] and expression shown as arbitrary units.

### Statistics

Differential protein expression was compared using repeated measures one-way analysis of variance (1-way RMANOVA) in the Biological Variation Analysis module in DeCyder 7 (GE Healthcare). p-values were corrected for multiple comparisons by the method of Benjamini and Hechberg [30] in R 2.14.0 [31] to yield false discovery rates (FDR) for the DIGE experiments. Significant differences in protein expression using immunodetection were compared using RMANOVA in R, with hibernation and reproductive status as categorical factors.

## Results

### Animals

The average Julian day of sampling were 301 and 58, respectively for PRE and HIB samples. All animals were adult females (mean age: 8.3 years), and five were pregnant at the time of den entry. All five gave birth during hibernation, and the average Julian day of parturition was 18. One cohort of cubs was lost shortly after birth (bear #5). A mean of 57.4 lbs or 27% of initial body weight was lost during hibernation, and no correlation was observed in this weight loss between pregnant and non-pregnant animals.

### Serum proteome

**Figure 1:**
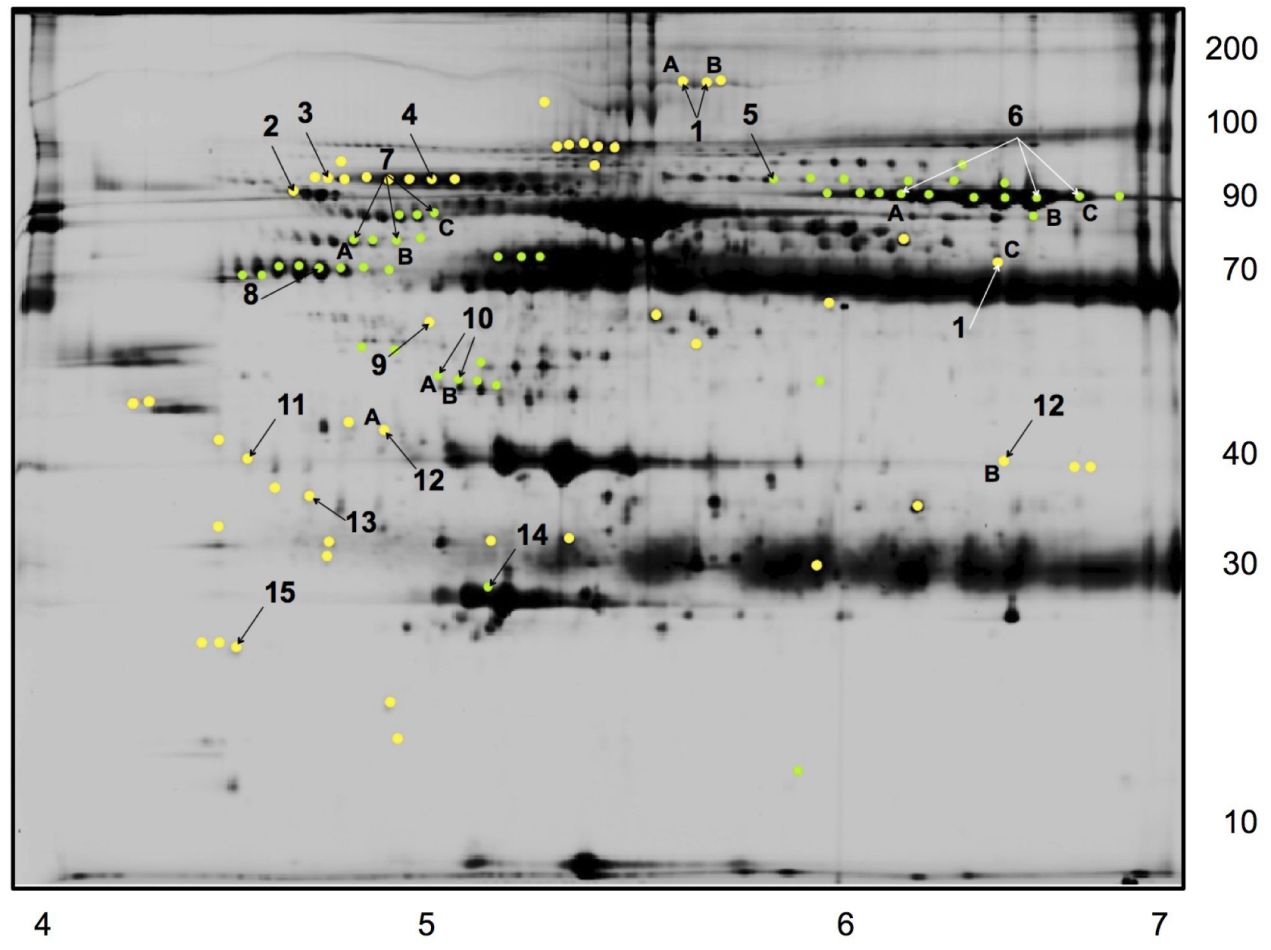
Bear serum proteomics. Representative images of a Cy2-stained black bear serum proteins separated by difference gel electrophoresis, utilizing a pH 4–7 (left to right) immobilized pH gradient isoelectric focusing gel strip for the 1^st^, horizontal separation and a 12% SDS-PAGE gel in the 2^nd^, vertical separation. Protein spots significantly (p<0.05) up-regulated (yellow) and down-regulated (green) proteins are indicated by colour. Protein spots that were subsequently identified by tandem mass spectrometry are indicated by numbers, which correspond to spot IDs in table 2. Estimated protein molecular weights in kiloDaltons (kDa) are indicated on the right edge of the image, and estimated isoelectric points are on the bottom edge.

**Figure 2:**
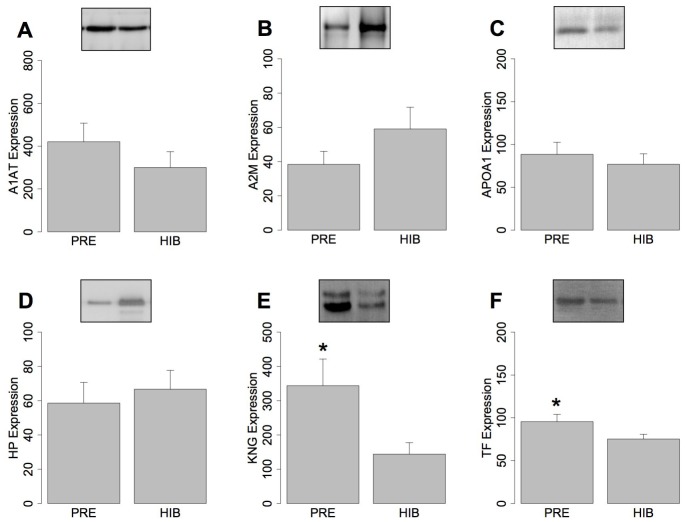
Specific proteins expression in bear serum. Western immunoblots and histograms of the mean + standard error of the mean (n = 8) protein expression (arbitrary units as a proportion of pooled reference black bear serum) between pre-hibernating (“Pre”) and hibernating (“Hib”) black bears using antibodies against A) α1-antitrypsin (A1AT), B) α2-macroglobulin (A2M), C) apolipoprotein A1 (ApoA1), D) haptoglobin (Hp), E) kininogen 1 (KNG), and F) transferrin (Tf). Changes in protein expression between hibernation states of individual bears are overlaid on top of the histogram. A representative western blot is shown inset, and the left and right wells are loaded with pre-hibernation and hibernation serum samples, respectively. Statistically significant differences (p<0.05, paired 1-way ANOVA) between hibernation states are indicated with asterisks. A2M was significant at p = 0.07.

The DIGE analysis identified a maximum of 2230 total protein spots per sample, of which 70 spots were differentially expressed in the hibernating bears. In total 36 and 34 protein spots were down- and up-regulated, respectively, in the HIB compared to the PRE samples (Figure 1 and Table 2). Many of these differentially expressed spots formed electrophoretic trains, suggesting multiple isoforms. The differentially expressed protein spots were ranked based on their p-values and the top 29 spots were chosen for identification by mass spectrometry. We obtained 23 protein IDs (Figure 2 and highlighted spots in Figure 1); all but two of the identified proteins were matched to giant panda (*Ailuropoda melanoleuca*) predicted proteins in the NCBI non-redundant protein database, and the remaining two were matched to dog (*Canis familiaris*) proteins. The identified proteins that were up regulated during hibernation (abbreviated gene names and accession numbers in parentheses) included α_2_-macroglobulin (A2M; EFB20759), α_1_B-glycoprotein (A1BG; EFB23492), complement components C1s (C1S; EFB13954) and C4 (C4; EFB21208), immunoglobulin μ heavy (IGHM; AAX73309) and J chains (IGJ; EFB23253), α_1_-antitrypsin (A1AT; XP_002920519), clusterin (CLU; EFB22766), and haptoglobin (HP; EFB23129). Down regulated proteins included C4b binding protein α chain (C4BPA; EFB13508), transferrin (TF; EFB18586), kininogen 1 (KNG; XP_002914859), α_2_-HS-glycoprotein (AHSG; XP_002914863), and apolipoproteins A-I (APOA1; XP_002919539) and A-IV (APOA4; XP_546510).

**Table 2:** Protein spots identified by tandem mass spectrometric analysis and MASCOT database searching.

Spot ID	Protein ID	Accession	Fold Change	p	FDR
1A	α2-macroglobulin	EFB20759	2.1	0.003	0.128
1B	α2-macroglobulin	EFB20759	1.7	0.004	0.130
1C	α2-macroglobulin	EFB20759	1.8	0.013	0.205
2	α1B-glycoprotein	EFB23492	1.5	0.021	0.263
3	Complement C1s subcomponent	EFB13954	1.6	0.001	0.128
4	Immunoglobulin μ Heavy Chain	AAX73309	1.6	0.002	0.128
5	C4b binding protein α chain precursor	EFB13508	−1.5	0.007	0.165
6A	Transferrin precursor	EFB18586	−1.8	0.011	0.192
6B	Transferrin precursor	EFB18586	−1.9	0.006	0.164
6C	Transferrin precursor	EFB18586	−2.1	0.004	0.130
7A	Kininogen 1	XP_002914859	−1.7	0.011	0.192
7B	Kininogen 1	XP_002914859	−2.5	0.005	0.147
7C	Kininogen 1	XP_002914859	−1.8	0.022	0.268
8	α2-HS-glycoprotein	XP_002914863	−1.9	0.024	0.195
9	Complement component C4	EFB21208	1.5	0.003	0.128
10A	Apolipoprotein A-IV	XP_546510	−5.7	0.003	0.128
10B	Apolipoprotein A-IV	XP_546510	−8.1	0.004	0.128
11	α1-antitrypsin	XP_002920519	1.6	0.024	0.268
12A	Clusterin	EFB22766	1.5	0.032	0.303
12B	Clusterin	EFB22766	2.0	0.004	0.139
13	Haptoglobin	EFB23129	2.0	0.007	0.168
14	Apolipoprotein A-I	XP_002919539	−1.7	0.042	0.323
15	Immunoglobulin J chain	EFB23253	1.5	0.044	0.328

Spot IDs correspond to labeled spots in Figure 1.

p-values from the 1-way RMANOVA and False Discovery Rate (FDR) for each protein are shown. Fold changes are relative change in protein spot volume from pre-hibernation to hibernation.

These identified proteins were grouped into Gene Ontology categories of biological function in GoMiner, and selected significantly enriched categories are presented in Table 3. Many of the proteins are multifunctional and involved in several pathways, including immune system processes (GO:002376: CLU, AHSG, KNG, C4, C1S, C4BPA, A2M, APOA1, IGHM, APOA4, and IGJ). These proteins can also be subcategorized as proteins involved in adaptive (GO:0002250) and innate immunity (GO:0045087), complement activation (GO:0006956), and the acute phase response (GO:0006953). Three other significantly enriched GO categories included digestion (GO:0007686: APOA1 and APOA4), platelet degranulation (GO:0002576: A2M, APOA1, CLU, KNG, A1AT, and TF) and the response to wounding (GO:0009611: CLU, AHSG, A1AT, APOA1, C4, KNG, A2M, APOA4, HP, and TF).

**Table 3:** Significantly enriched (Enrichment >1.5, p<0.05) selected Gene Ontology categories of serum proteins in hibernating black bears.

Category Name	GO Category ID	p-value	FDR	Enrichment	Changed Proteins in Category	Total Proteins in Category
Immune System Process	002376	0.0036	0.0000	1.98	11	79
Adaptive Immune Response	0002250	0.0111	0.0562	3.23	5	22
Innate Immune Response	0045087	0.0212	0.1018	2.43	6	35
Acute Phase Response	0006953	0.0001	0.0000	6.09	6	14
Complement Activation	0006956	0.0275	0.2000	2.63	5	27
Response to Wounding	0009611	0.0083	0.0556	1.95	10	73
Digestion	0007586	0.0257	0.1791	7.10	2	4
Platelet Degranulation	0002576	0.0035	0.0000	3.41	6	25

The False Discovery Rate (FDR) and number of changed and total proteins in each GO category are shown.

### Immunodetection

Differential expression of proteins identified using DIGE was confirmed by immunodetection of select serum proteins in the bears prior to and during hibernation. We performed western immunoblotting on an expanded set of black bear serum samples collected during the PRE and HIB periods using antibodies against 6 of the proteins identified in our MS/MS analysis (Figure 2). We found that pregnancy status had no significant effect on the expression of any of the tested proteins. KNG (F = 10.7, p<0.05) and TF (F = 10.4, p<0.05) were significantly reduced in HIB compared to PRE bears and this is in agreement with our DIGE results (Table 2 and Figure 2E and F). Also, A2M expression was higher (F = 3.22, p = 0.07) in the HIB bears compared to PRE animals (Fig. 2B). There were no significant differences in A1AT, APOA1, and HP expression in HIB compared to PRE bears (Figure 2A, C, and D).

## Discussion

Our results demonstrate for the first time the changes in the serum proteome that occur during hibernation in the American black bear. Only a small proportion (<5%) of serum proteins were differentially regulated according to our selection criteria during hibernation compared to active black bears, and of these equal numbers of proteins were either up- or down-regulated. In comparison, studies on gene expression changes in the liver of black bears found that approximately 7% of all genes were differentially expressed (using our selection criteria), and 67% of these differentially expressed genes were up regulated during hibernation [17]. We hypothesize that changes in specific serum proteins, including those involved in immunity, coagulation, and bone metabolism, are essential in maintaining the hibernation phenotype and health in black bears.

Immunity-related processes were affected by hibernation, and this was evident based on the protein categories enriched in the serum of hibernating black bears. Prolonged fasting and hypothermia in animals generally has suppressive effects on immunity, including the atrophy of lymphoid tissues, suppressed reactions to antigens, and leukopenia [32], [33], [34]. In other hibernators, including sciurids, both the adaptive and innate immune systems exhibit reduced functionality during bouts of torpor [35]. The differences in core body temperature between hibernating sciurids (near freezing) and black bears (30–36°C) may be a reason for this difference as the suppression of immune function is associated with the degree of hypothermia [32]. During the hibernation season, squirrels frequently arouse from torpor to warm up to normothermic body temperature for 5–24 h before re-entering torpor [1], [36]. However, despite the large energetic costs associated with arousals in sciurids, the frequent episodes of near normothermia are associated with bursts of immune function essential to clear the pathogen loads that may have accumulated during the torpor bout [35], [37].

Bears have a prolonged hibernation period, unlike the 10–14 day bouts of hypometabolism in sciurids that are interrupted by brief (5–24 h) interbout arousal periods. Thus, ursids may have adopted different strategies to cope with this unusual physiological state of metabolic depression. The differential regulation of immunity-related proteins during hibernation may be one such adaptation that allows bears to remain in their hypometabolic and mildly hypothermic state, while aiding in the maintenance of immune competence and resistance against infection and disease. Moreover, a recent report demonstrated that the healing of cutaneous wounds is maintained during hibernation [7], suggesting that the mechanisms underlying wound healing and immune function are active during hibernation in bears. This is a unique adaptation because hypothermia and metabolic depression suppresses wound healing in other animals [12], [13]. Since the initial response to wounding includes the participation of the innate immune system in restoring hemostasis and preventing the development of infections [38], our results suggest that serum immune-related protein changes may be playing a key role in this unique healing ability of black bears during hibernation. Moreover, it is unlikely that the differential expression of these immune proteins were induced by an immune response since A2M and HP have been shown to be upregulated during hibernation independent of inflammatory acute phase reaction in brown bears [20]. While stress may enhance acute phase response in animals [39], we did not observe any behavioral changes associated with handling during blood collection in bears arguing against stress as a possible factor in the observed acute phase response in hibernating bears. In general, however, the immune system of ursids is presently poorly characterized, and it remains to be determined how the changes in protein expression that were observed in this investigation translate to the functioning of the immune system during hibernation.

Other proteins that were modulated by hibernation, including A2M and KNG, appear to play roles in the regulation of blood coagulation. The blood of hibernating animals have been observed to be in a hypocoagulable state, including in squirrels and hedgehogs [40], [41], and platelet aggregation has been shown to be reduced in brown bears several days after arousal from hibernation [42]. This decrease in coagulation activity may contribute to the prevention of blood clotting in the face of low cardiac output and increased blood viscosity during hibernation [2]. Elevations in serum A2M levels during hibernation in squirrels have been linked to increases in clotting times [43]. The down regulation of KNG expression during hibernation may also contribute to this state of hypocoagulation as this protein is a coagulation cascade cofactor that increases the rate of some enzymatic reactions, including prekallikein to kallikein and factor XI and XII to XIa and XIIa, respectively [44], [45]. Moreover, decreased body temperatures during hibernation and in hypothermic states may be playing a role by reducing coagulation enzyme activity and platelet adhesion, which may also contribute to increased clotting times [40], [46]. Based on our serum proteome data, we propose that coagulation activity and blood clotting may be reduced in hibernating bears.

Hibernating black bears preserve bone mass despite several months of inactivity and anuria [15], and the mechanisms underlying this phenomenon has been the subject of intense research due to its possible implications for human medicine. Bone remodeling during hibernation in bears decreases to approximately 25% of summer active levels and bone formation and resorption are balanced [8]. Other hibernators exhibit increased osteoclastic and decreased osteoblastic activity [14], [47], resulting in overall decrease in bone mass during torpor. Additionally, markers of bone resorption and formation, including ICTP [48], CTX [49], and osteocalcin [50] are elevated during hibernation in black bears, but these markers may accumulate due to a lack of renal clearance rather than increases in the rates of bone remodeling. We did not detect these serum markers of bone metabolism in this study and this may be because low molecular weight peptides (e.g. osteocalcin: 5.8 kDa [50], ICTP: 9 kDa [48], CTX: 2 kDa [49]) are generally poorly resolved in 2D gels. However, we found that serum AHSG levels were down regulated during hibernation in bears. AHSG is secreted by the liver into circulation, where it is a major carrier protein of calcium phosphate and carbonate, and is a major non-collagen protein constituent of bone [51]. This protein has been implicated as an inhibitor of osteogenesis [52] and may regulate bone remodeling by binding to and blocking the action of cytokines that modulate bone marrow cell proliferation and mineralization by the transforming growth factor (TGF)-β family of cytokines [53]. The targeted deletion of one copy of the AHSG gene in mice resulted in a two-fold decrease in serum AHSG levels and abnormal bone development, including decreased mineral formation rate and increased mineral content [53], which may suggest a role for this protein in modulating the rate of bone formation. Thus, we propose that the down regulation of AHSG in hibernating black bears may lead to a reduction of TGF-β antagonist activity and the modulation bone formation rates. Furthermore, the immune system plays an important role in bone remodeling [54], and the changes in immune-related proteins in black bear serum during hibernation suggests a role for these protein changes in bone remodeling. However, further research is warranted to elucidate the specific proteins and processes involved and their modes of action.

We found that A1BG is up regulated during hibernation in bears. A1BG is a homologue of the woodchuck hibernation induction trigger (HIT), which has been proposed as a putative cross-species hibernation inducer by acting on opioid receptors [55]. There have been reports of successful induction of hibernation in active animals by injection with purified serum containing HIT [56], but other attempts to reproduce this effect in other species have not been successful [57]. HIT possesses properties similar to that of the delta opoid receptor agonist [D-Ala^2^, D-Leu^5^]-enkephalin (DADLE) [58] and this opioid activity can protect organs against ischemia-reperfusion injury [59], [60]. However, the mechanisms underlying the actions of this protein remain unclear, but the up regulation of A1BG during hibernation in black bears suggests that this opioid activity may be an adaptation to protect tissues and organs during hibernation, including hypothermia and reduced blood flow.

There were other protein changes during hibernation, including possible markers of nutritional status, but their functions are poorly characterized or have never been studied in bears. For instance, APOA1 [61], APOA4 [61], [62], and TF [63] have been implicated as markers of fasting in other species, and their down regulation in the serum of hibernating, anoretic black bears supports these proteins as markers of fasting status in animals. Additionally, these proteins are involved in various facets of peripheral nutrient transport [64], and increases in serum APOA4 levels has also been implicated as a putative satiety signal [65]. Together, the down regulation of these proteins may reflect a lower metabolic capacity and nutrient transport during hibernation in black bear.

The mechanisms underlying the changes in the hibernating serum proteome of bears are largely unclear. We cannot rule out if other factors played a role in the observed changes. For instance, recently, Seger and coworkers found that levels of some markers of bone metabolism were different between lactating and non-lactating hibernating black bears [49], and suggested that the metabolic demands of lactogenesis were associated with increased bone resorption. In the present study, our sample size was not large enough (n = 4) to assess whether lactation influenced the hibernating serum proteome of black bears. However, we did not observe any significant effect of reproductive status on candidate protein expression in our immunodetection analysis, arguing against lactation as a factor. Changes in protein levels may also be due to either decreased excretion, associated with anuria in bears [66], or increased secretion of proteins into the serum. To this end, the differential expression of some serum protein genes in the liver of hibernating black bears have been reported, including α_2_-HS-glycoprotein, clusterin, and α_2_-macroglobulin [17], [67]. Future studies should be designed to elucidate the mechanisms underlying these serum proteome changes in hibernating black bears.

In conclusion, for the first time we demonstrate that hibernation in black bears is associated with differential expression of serum proteins involved in immunity, coagulation, and bone metabolism. Furthermore, these results suggest novel mechanisms for some of the unique and remarkable metabolic and physiologic attributes of hibernating black bears, including the maintenance of wound healing [7], which contrasts with the lack of wound healing in other species during fasting and hibernation [35]. The differential expression of immune-related proteins during may confer a survival advantage by enhancing processes that prevent infections and diseases during prolonged hibernation in black bear. We also identified differentially expressed proteins that were associated with fasting, coagulation, and bone remodeling leading us to hypothesize a key adaptive role for these proteins in maintaining the health of hibernating black bears.
